# Radiation safety: time to act

**Published:** 2017

**Authors:** SC Brown

**Affiliations:** Department of Paediatric Cardiology, University of the Free State, Bloemfontein, South Africa

## Introduction

It is an acknowledged fact that interventional cardiologists have the highest occupational radiation exposure of all medical professionals. As a matter of fact, interventional cardiac procedures represent the largest contribution of ionising radiation source after computerised tomography and nuclear medicine. Modern therapies and the need for quality radiological imaging have dramatically increased the use of ionising radiological imaging in cardiology.

Radiation safety is rapidly becoming an important issue. The first major drive towards this goal gave rise to the establishment of the international radiation protection association (IRPA) in late 2002, leading to the publication of guiding principles for establishing a radiation-protection culture.^1^ The aim of such a culture is to substantially reduce radiation dose to both patients and staff.

## Biological effects of radiation

It should be taken into account that patients, technicians, nurses and cardiologists are at risk of these effects. There are two categories of unwanted effects when exposed to ionising radiation:

Deterministic effects: here an identifiable threshold level exists and the severity of effect intensifies with increasing dosage of exposure. Biological effects occur as a result of cell damage and death. Symptoms are related to the extent of cell death. Dermatological effects and cataracts are typical examples of deterministic effects.
Stochastic effects: these follow a linear non-threshold theory, which essentially means these effects occur by chance. There is no minimum exposure, and risk increases linearly with radiation dose received. Cancer in an exposed individual occurs due to the mutation of cells as a result of chromosomal translocations.

## Health hazards

Cataracts: posterior sub-capsular cataracts have been reported in 50% of cardiologists and 41% of nurses working in interventional catheterisation laboratories.^2^ The authors observed that lens changes were associated with several years of work without eye protection and cumulative doses were in the range of 0.1 to 18.9 Sv.
Brain tumours: several case reports of brain tumours have emerged in the literature and have occurred in more than 31 physicians working in catheterisation laboratories, mostly interventional cardiologists.^3^-^5^ Of particular interest is the fact that up to 85% of brain tumours were left sided – the area of the head closest to the X-ray tubes. The physicians in this report were exposed to ionising radiation over a period of 12 to 30 years.
Other: thyroid changes and neoplasms, hypertension, hyperlipidaemia, reproductive and even psychological effects have been described.^6^-^8^ Hair loss and skin damage may follow prolonged exposure during fluoroscopic procedures. These vary from temporary erythema to necrosis of the skin and subcutaneous tissues. A single dose of 6–8 Gy on a 5-cm2 field may trigger tissue damage.^9^ It should be noted that the hands of operators receive the highest exposure during cardiac interventions.

## Food for thought

The article by Rose et al. (page 196) in this edition of the journal gives a sobering perspective on radiation protection in South Africa.^10^ The study included public- and private-sector radiologists and cardiologists. It is obvious from the results that a complacency and lack of knowledge regarding radiation safety is prevalent among cardiologists.

In essence, the results show that little or no formal education for cardiology fellows regarding radiation protection is offered during training. Even more disconcerting is the fact that even though heads of units (both adult and paediatric cardiology) acknowledged the need for radiation safety measures and training, precious little appears to be done to address the issue. This is compounded by the fact that junior fellows expressed concerns regarding the effects of radiation exposure on their long-term health, and that only one question regarding radiation safety appeared in the national exit examinations for cardiologists.

## What shoud be done

It is mandatory to establish a radiation safety culture for cardiologists. Basic training should be available for all healthcare workers in the catheterisation laboratory, and ongoing radiation safety courses should be obligatory. Unless training units actively promote and examine fellows on radiation safety, little will change.

Simple precautions to minimise exposure to patients, staff and operators should be instituted as enshrined in the ALARA (as low as reasonably achievable) principles. The American Heart Association statement on enhancing radiation safety in cardiovascular imaging may be followed as a guideline – clear strategies and action plans to reduce exposure to patients and staff should be followed.^11^ Institutional insensitivity should also be addressed and the proper fundamental principles of radiation protection should be rigidly applied.

The profession should be concerned about how interventionists and young cardiologists with long careers ahead of them can avoid the ravages of exposure to ionising radiation. Over a lifetime, how much radiation exposure is acceptable and how much are we at risk of the complications of prolonged and recurrent exposure? The time to act is now.
